# Limits of Structural Plasticity in a Picornavirus Capsid Revealed by a Massively Expanded Equine Rhinitis A Virus Particle

**DOI:** 10.1128/JVI.01979-13

**Published:** 2014-06

**Authors:** Saskia E. Bakker, Elisabetta Groppelli, Arwen R. Pearson, Peter G. Stockley, David J. Rowlands, Neil A. Ranson

**Affiliations:** Astbury Centre for Structural Molecular Biology, University of Leeds, Leeds, United Kingdom

## Abstract

The Picornaviridae family of small, nonenveloped viruses includes major pathogens of humans and animals. They have positive-sense, single-stranded RNA genomes, and the mechanism(s) by which these genomes are introduced into cells to initiate infection remains poorly understood. The structures of presumed uncoating intermediate particles of several picornaviruses show limited expansion and some increased porosity compared to the mature virions. Here, we present the cryo-electron microscopy structure of native equine rhinitis A virus (ERAV), together with the structure of a massively expanded ERAV particle, each at ∼17–Å resolution. The expanded structure has large pores on the particle 3-fold axes and has lost the RNA genome and the capsid protein VP4. The expanded structure thus illustrates both the limits of structural plasticity in such capsids and a plausible route by which genomic RNA might exit.

**IMPORTANCE** Picornaviruses are important animal and human pathogens that protect their genomic RNAs within a protective protein capsid. Upon infection, this genomic RNA must be able to leave the capsid to initiate a new round of infection. We describe here the structure of a unique, massively expanded state of equine rhinitis A virus that provides insight into how this exit might occur.

## INTRODUCTION

**FIG 1:**
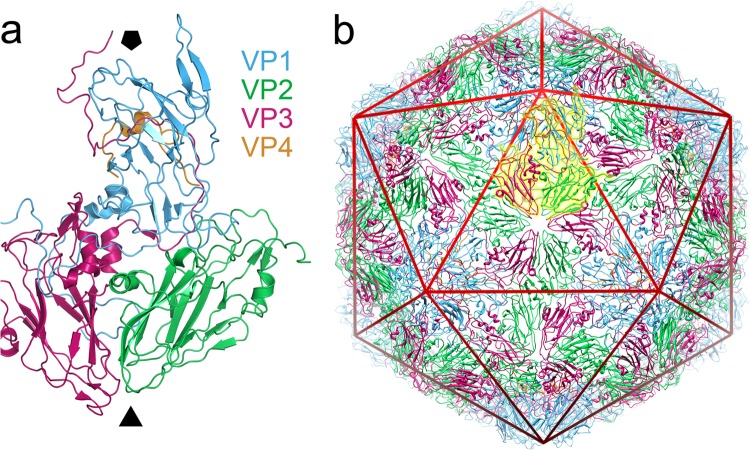
Structure of equine rhinitis A virus. (a) X-ray structure of the native equine rhinitis A virus particle. A single asymmetric unit of the native ERAV virion (PDB 2WFF) is shown in diagram representation, from a view outside of the viral capsid. The viral proteins are colored blue (VP1), green (VP2), magenta (VP3), and orange (VP4), and the approximate position of the viral 5- and 3-fold axes are indicated by the solid black pentagon and triangle, respectively. The color coding of VP1 to VP4 is identical in all figures in the manuscript. (b) The intact, pT=3 capsid structure. The asymmetric unit shown in panel a is highlighted in yellow, and a icosahedron is included as a red wire mesh. The image was produced using PyMOL (www.pymol.org) and UCSF Chimera (35).

The picornaviruses are a family of nonenveloped viruses with a relatively small (∼300 Å in diameter; see Fig. 1a) icosahedral capsid and a positive-sense, single-stranded RNA genome (1, 2). The family contains important pathogens of a wide range of species and have enormous consequences for human health and the agricultural economy. Picornaviruses that cause notable diseases in humans include poliovirus, enterovirus 71 (EV71), rhinoviruses A to C, and hepatitis A virus. In animals, there is increasing evidence that picornavirus-like viruses such as deformed wing virus are partly responsible for declining honey-bee populations and are thus a major threat to food security (3), while foot-and-mouth disease virus (FMDV) is arguably the most economically important pathogen of agricultural livestock. Equine rhinitis A virus (ERAV) is closely related to FMDV. Both ERAV and FMDV are classified in the genus Aphthovirus (4), owing to similarities in their genome sequences (5–7) and the physicochemical properties of their capsids (8, 9). ERAV causes febrile respiratory tract infections in horses that resemble the symptoms of the common cold (10). It infects a broad range of cell types and, like FMDV, it causes viremia and persistent infections (6). ERAV is now being used as a model system to investigate the biology of an FMDV-like virus without the prodigious challenges of working in the stringent biocontainment needed for FMDV itself.

A nonenveloped, single-stranded RNA virus such as ERAV faces a formidable challenge in getting its genome into the cytoplasm of its host cell, where translation of viral proteins and replication of the viral genome can begin. Recently, we have shown that ERAV, like FMDV, enters the host cell in a clathrin-dependent manner and that infection is dependent on endosomal acidification (11). However, it remains unclear how the structure of a picornavirus capsid, together with triggers such as a change in pH, orchestrates the translocation of the RNA genome across the lipid bilayer of an endosomal membrane into the cytoplasm (for a review, see reference 12).

The generalized picornavirus capsid is built from 60 copies of each of four structural proteins (VP1 to VP4; Fig. 1a) (13–15), which are produced by cleavage of a single polyprotein by viral proteases (for a review, see reference 16). VP1 to VP3 each comprise an eight-stranded beta sandwich and form the three quasi-equivalent conformers required to build a capsid with a pseudo-T=3 surface lattice (13, 14). The molecular mechanisms by which the genome is delivered from this protective capsid into the cytoplasm of host cells remain poorly understood. The virus for which this is best characterized is poliovirus, a member of the Enterovirus genus, in which binding to a cell surface receptor initiates conformational changes resulting in a change from the mature 160S particle to a 135S form which has lost the internal protein VP4 and externalized the hydrophobic N terminus of VP1. The 135S particle is then converted by an unknown mechanism to an empty 80S particle from which the genome has been ejected (for a review, see reference 2). For poliovirus, it has been shown that the native state is structurally dynamic, undergoing “breathing” motions that allow reversible externalization of the VP1 N terminus (17).

A great deal of structural information is available for picornavirus capsids and their intermediates. Recent work on EV71 (18, 19) has compared structures for mature (150S) and empty (82S) particles that show slight (∼4 to 5%) but significant expansion. This structural rearrangement generates small holes near the base of a surface depression known as the “canyon” and a somewhat larger hole at the icosahedral 2-fold axes. However, neither of these holes appears to be large enough to readily allow RNA egress. Further conformational change could lead to merging of the holes to form a larger pore that could allow uncoating and a slightly larger (∼10 Å) pore at the 2-fold axis has been reported recently (20, 21). This would accord with previous suggestions, based on cryo-electron tomography, suggesting that RNA leaves poliovirus near a 2-fold axis (22), and is able to cross a membrane (23). For ERAV, which is very similar in structure to FMDV (15, 24), the X-ray structures of both the native virion and low-pH-induced empty particle are extremely similar, with a root mean square deviation between the structures of only ∼0.9 Å (15). Thus, there is little appreciable expansion of the particle and no obvious exit route by which the genomic RNA could have left. In contrast to enteroviruses, such as poliovirus, the end products of the aphthovirus uncoating process are not empty capsids but dissociated pentameric subunits (15, 25). However, based on X-ray crystal structures and biochemical evidence, it has been suggested that genome transfer during the infection process proceeds via a transient empty particle (15). The details of the structural transitions that might allow the genome to leave the ERAV capsid are even less well understood than for poliovirus.

Here we report two structures of ERAV that together provide a fascinating insight into just how large structural transitions in picornavirus capsids can be and give a potential route for RNA to leave. Using cryo-electron microscopy (cryo-EM) and single particle image processing, we have determined the structure of native ERAV virions, together with the structure of a massively expanded, empty ERAV particle with large pores through the capsid layer.

## MATERIALS AND METHODS

### Cryo-EM data collection and image preprocessing.

Native ERAV was purified as described previously (15) and stored in a sucrose containing buffer (50 mM NaCl, 50 mM HEPES [pH 7.3], ∼30% sucrose) at 4°C. Samples were then dialyzed against phosphate-buffered saline overnight, and spin-concentrated before use. Next, 3-μl aliquots of ERAV at ∼2 mg ml^−1^ were applied to 300-mesh Quantifoil R1.2/1.3 EM grids that had been glow discharged in air for ∼30 s immediately before use. Grids were frozen by plunging into liquid nitrogen-cooled, liquid ethane, using a computer-controlled, pneumatically driven freezing apparatus (26). Grids were imaged on an FEI Tecnai-F20 microscope equipped with a Gatan 626 cryo-transfer stage, using low-dose protocols (∼15 e^−^/Å^2^). Images were recorded on a Gatan US4000SP charge-coupled device camera a calibrated magnification of ×87,209, giving a final object sampling of 1.72 Å/pixel. Micrograph defocus and astigmatism were determined computationally using the program CTFFIND3 (27), and micrographs showing significant drift or astigmatism were discarded. Small and large particles were readily distinguishable in the raw micrographs, and all particles recognizable as either small or large were interactively selected using the program BOXER (28) and corrected for the microscope contrast transfer function by computational flipping of image phases in SPIDER (29). Judging the relative proportion of large and small particles in solution is problematic, as the cryo-EM data set necessarily only contained isolated, discrete particles, and the large particles were more prone to clumping. The final data sets contained 822 small (55%) and 663 large (∼45%) particles, but this probably underestimates the proportion of large particles present in solution. All other image-processing steps were performed in SPIDER. All image data were band-pass filtered between 350 and 6 Å and normalized to a constant mean and standard deviation.

### Single particle reconstruction.

**FIG 2:**
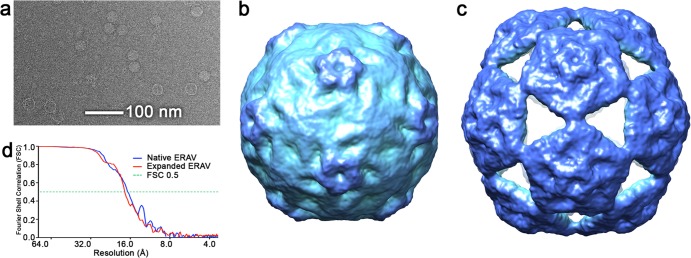
Solution structure of native and expanded ERAV particles. (a) Raw cryo-electron micrograph of the ERAV sample. Two types of particle—small and large—are clearly visible in the raw data. (b) Cryo-EM structure of small ERAV particle, which corresponds to the structure of the native virion. (c) The corresponding structure of the large, expanded ERAV particle. For Fig. 2, 3, and 5, the cryo-EM-derived density for both native and expanded particles is colored with the radial color scheme described in Fig. 5. (d) Fourier shell correlation plot for both EM structures. The resolutions of the maps are 16.9 Å for the native structure and 17.4 Å for the expanded particle (both using the FSC0.5 criterion).

Single-particle refinement and three-dimensional (3-D) reconstruction were carried out essentially as described previously (30). The atomic coordinates for wild-type poliovirus (Mahoney strain; PDB ID 1ASJ7 [31]) were converted to density and all high-resolution features removed by Fourier filtration to 40 Å using a low-pass Fermi filter. The resulting low-resolution, RNA-free model of a generic picornavirus capsid was then used as a starting model for EM structure refinement of both data sets. The starting model was projected across 78 orientations evenly covering the icosahedral asymmetric unit with a spacing of 3°. All image data were then aligned to these views. After alignment, averages of the images corresponding to each view were calculated. 3-D reconstructions were generated after each refinement round using weighted back-projection and imposing icosahedral symmetry. The resulting reconstruction was then used to generate a new series of reference views, and the whole procedure was iterated. In the final rounds of refinement, images corresponding to each reference orientation were ranked according to cross-correlation coefficient and the worst-aligning members for each orientation were excluded. The final reconstructions included 260 of 822 particles and 227 of 663 particles for the native and expanded structures, respectively. The resolution of each map was determined by Fourier shell correlation (FSC) between reconstructions calculated from half of each data set and using the FSC = 0.5 criterion (Fig. 2d), which indicated resolutions of 16.9 Å for the native, and 17.4 Å for the expanded state.

### Accession codes.

The cryo-EM maps of native and expanded ERAV have been deposited under Electron Microscopy Data Bank accession codes EMDB-2389 and EMDB-2390, respectively. The fitted atomic coordinates for the native particle have been deposited under Protein Data Bank (PDB) accession code 4CTF and for the expanded particle under 4CTG.

## RESULTS AND DISCUSSION

The two structures reported here were solved from a single cryo-EM data set. The ERAV used was expressed in Ohio HeLa cells, purified by sucrose-gradient fractionation, and stored in a sucrose-containing buffer at 4°C (15). When we examined images of what we expected to be native ERAV particles, it was immediately apparent that two distinct forms were present: one small and one large (Fig. 2). A data set of each type (822 small and 663 large) was collected, and a structure for each was determined using single particle image processing and icosahedral averaging as described previously (30). The results were 3-D structures for two very different ERAV particles. The smaller particles generated a structure that, at least at the intermediate resolution of our map, is indistinguishable from previous X-ray structures of mature ERAV (Fig. 1b) (15), while the large particles generated an expanded structure unlike any seen for any picornavirus capsid to date (Fig. 1c). The expanded capsid is much larger than the native state, with a maximum diameter (measured from the center of the particle to the tip of a pentameric turret) some 12% larger (355 Å versus 316 Å).

**FIG 3:**
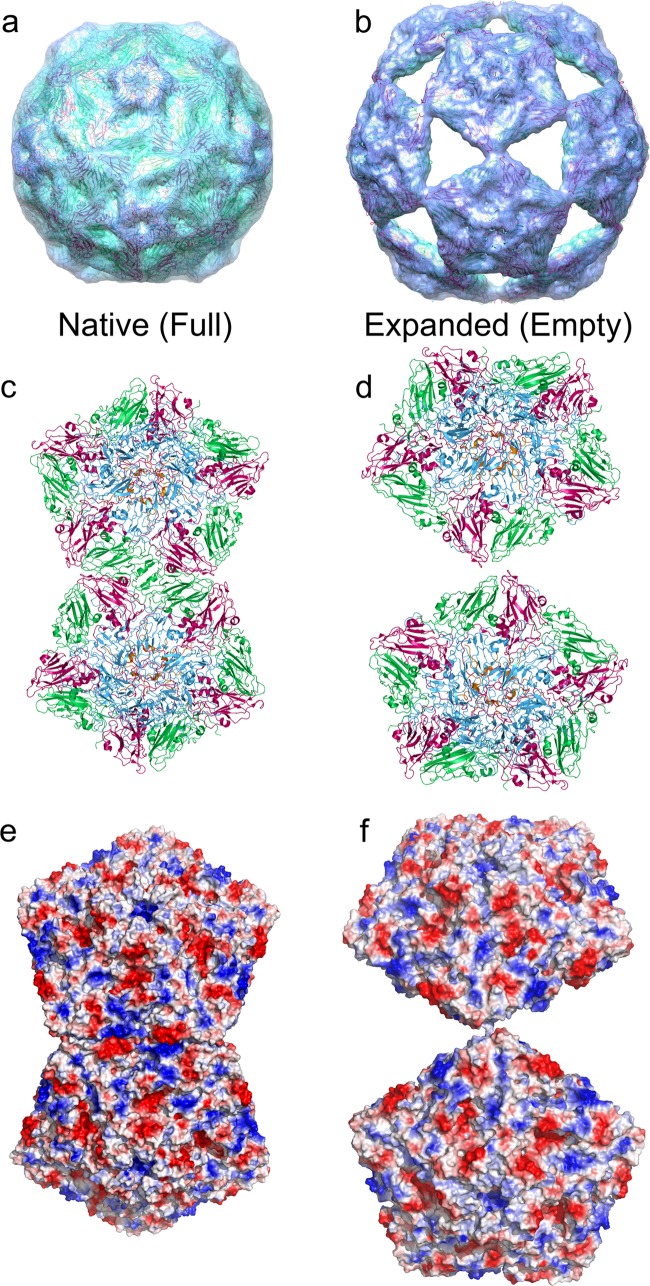
Structural transition required to reach the expanded state. The cryo-EM structures of the native (a) and expanded (b) ERAV particles with fitted atomic coordinates derived from the native particle structure (2WFF) are shown. The intact virion structure is fitted into panel a without adjustment, whereas the structure of an unmodified pentamer of VP1 to VP4 is fitted into panel b as a rigid body. The EM density is shown as a semitransparent surface, colored as in Fig. 2, and the fitted X-ray coordinates are shown in diagram representation and colored as in Fig. 1. (c and d) Close-up views of the interaction between two adjacent pentamers in the native and expanded particles. The view is down a viral 2-fold axis as in panels a and b. (e and f) Electrostatic surface potential of the two pentamers shown in panels c and d (calculated using the APBS plugin in PyMOL).

To try to understand how the ERAV capsid structure can accommodate such a large conformational change, we fitted the atomic coordinates for native ERAV (PDB ID 2WFF [15]) into the EM density of both the native and the expanded EM structures. As expected, the coordinates fit into our native (small) structure extremely well as a single intact unit (Fig. 3a), confirming that the smaller structure is of the native virion. However, the expanded state is clearly built from pentamers of the viral coat proteins, and a reasonable fit of the atomic coordinates could only be achieved as 12 isolated pentameric units (Fig. 3b). This is consistent with previous suggestions that the picornavirus capsomere is a pentamer (32).

**FIG 4:**
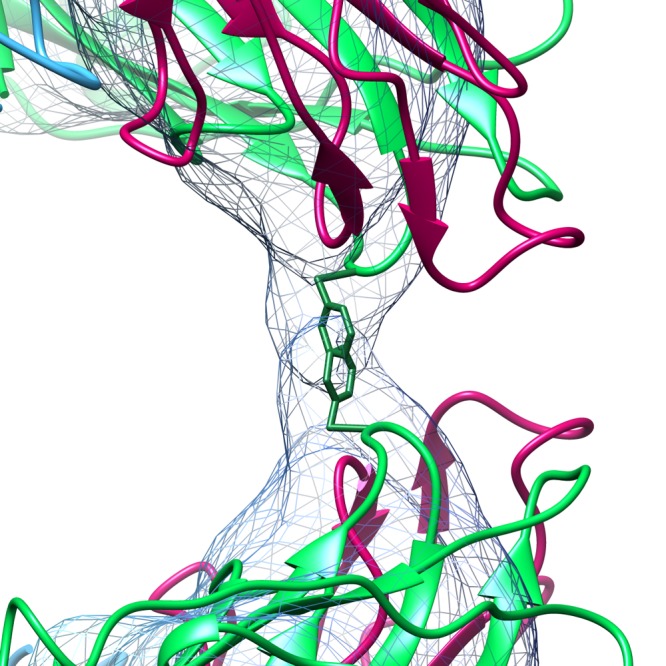
Contact holding the expanded particle together. The contact point between adjacent pentamer of VP1 to VP4 in the expanded particle lies across the 2-fold axes. At 2.75 σ, this contact in the expanded map is a discrete bridge of density that is occupied by Phe118 (shown as sticks) from each of the two symmetry-related copies of VP2 at the 2-fold axis. VP1 to VP4 are colored as described in the legend to Fig. 1.

The rotation and translation of the pentameric capsomeres required to form this particle from the native state is enormous, with the points of the pentons moving by as much as ∼60 Å in three dimensions. Unsurprisingly, such large movements completely change the interacting surfaces between all adjacent pentamers (Fig. 3c and d), disrupting a large interface with complementary electrostatic interactions (Fig. 3e and f) in the process. The new interface that holds the expanded particle together is obviously much smaller than in the intact particle. The core of the interaction appears to be predominantly hydrophobic in nature, with the strongest density for interpenton contacts in the expanded EM map corresponding to the location of PHE118 in VP2. Presumably, a ring stacking interaction occurs between the PHE118 residues of two VP2 molecules across the 2-fold axes of the particle (Fig. 4).

**FIG 5:**
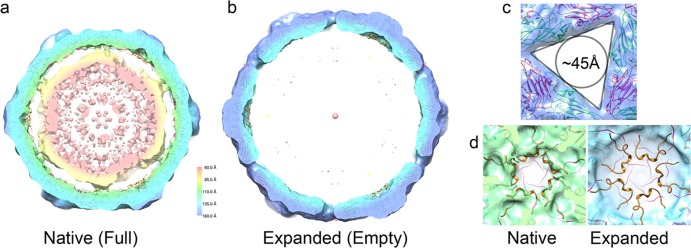
Internal differences between native and expanded ERAV particles. (a) 40-Å-thick central section through the cryo-EM structure of the native ERAV particle. The particle is filled with density that we ascribe to the encapsidated genomic RNA. (b) At an equivalent contour level, the expanded particle is almost entirely empty. (c) The pores on the 3-fold axis of the expanded state are large and triangular, with an in-circle diameter of ∼45 Å. (d) The internal surfaces of the native (left) and expanded (right) particles are very different. No density that can accommodate VP4 is present in the expanded particle, suggesting that it is not present in the expanded state. The crystal structure of the ERAV coat protein pentamer is docked into each EM map and is shown in a diagram representation colored as described in the legend to Fig. 1.

The energetic driver for such a dramatic remodeling of the pentamer interfaces is unclear. However, it is tempting to speculate that the polyanionic genomic RNA must play a role, and this seems plausible given the amounts of electron density for encapsidated RNA seen in the two structures. Shown in Fig. 5a is a central, 40-Å-thick slab through the structure of the native ERAV particle, which shows that the volume encapsidated by the protein shell is full of density that we attribute to the ∼8-kb single-stranded genomic RNA packaged in the mature virion. The expanded structure by contrast is almost completely empty at an equivalent contour level (Fig. 5b). The ∼12% expansion of the expanded particle leads to an ∼42% increase in encapsidated volume, so we would expect to see less electron density for RNA owing to lower degree of order and confinement. However, given the almost complete absence of density within the capsid, the genomic RNA appears to have left the particle. The pores in the expanded capsid are the obvious route by which RNA could leave. They are located at the icosahedral 3-fold axes, and are essentially triangular, with an edge of ∼75 Å and an in-circle diameter of ∼45 Å (Fig. 5c), easily enough to allow even base-paired regions of the RNA (with a diameter of 18 to 24 Å) to leave.

The lack of density for an encapsidated genomic RNA is not the only difference observed inside the capsids of the native and expanded states. When the inside surface of the pentameric capsomere of the expanded state is examined, it is clear that although a pentamer of VP1 to VP3 fits the EM density well, the underside of the ERAV capsomere has a broad, circular depression in the electron density. This corresponds precisely to the location of VP4 in the crystal structure, which is completely outside the envelope of the EM map. This region is very different to the density in the native particle (Fig. 5d). This strongly suggests that VP4 is not present in the expanded particle. The loss of VP4 from enterovirus capsids is known to precede genome uncoating (33), and there is circumstantial evidence that the same is true for aphthoviruses (34). This strongly suggests that the structure presented represents an uncoating intermediate rather than a reassembly of previously dissociated pentamers. Indeed, such reassembly, from isolated pentameric association of VP1 to VP3, has not previously been observed for any picornavirus capsid although poliovirus pentamers containing VP0, the uncleaved precursor of VP2 and VP4 can reassemble into empty particles (15). We have thus far been unable to reproduce the expanded state in the quantities required for either biophysical or more detailed structural analyses, although a small number of virus particles appear to be in the expanded state in all subsequent imaging experiments, even of fresh native virus.

The two structures presented here were serendipitously discovered after storage in a sucrose-containing buffer. We currently have no direct evidence that the expanded state is an “on pathway” intermediate in the normal uncoating process, although this is a priority for further experiments. However, we have demonstrated the conversion of virions into empty particles when exposed to conditions resembling those encountered during the early stages of endocytosis when cell entry occurs (15). The available structural evidence for picornaviruses of the enterovirus genus seems to be building toward a consensus describing pore formation near to a 2-fold axis (20, 22, 23). This is obviously different from the location of the pores in the expanded ERAV capsid described here at the 3-fold axes. However, the structure represented here may represent the endpoint of a structural transition that starts with such a 2-fold pore opening at a single location in the capsid.

Regardless of the functional importance of the expanded structure in the normal ERAV uncoating process, conformational changes of this magnitude are unprecedented in the picornaviruses. The structure presented here will change our ideas about how just how dynamic picornavirus capsids can be and how large a structural transition might be accommodated and still retain a recognizable capsid structure. This will perhaps also change our view about the transient states that could be accessible during picornavirus uncoating reactions *in vivo*.
